# Decoding B Cells in Autoimmune Diseases Through ScRNA + BCR-Seq: Current Knowledge and Future Directions

**DOI:** 10.3390/cells14070539

**Published:** 2025-04-03

**Authors:** Kai Quan, Huifang Wang, Peng Su, Yuanyuan Xu, Xinsheng Yao

**Affiliations:** Department of Immunology, Center of Immuno-Molecular Engineering, Innovation & Practice Base for Graduate Students Education, Zunyi Medical University, Zunyi 563002, China; 19191301316@163.com (K.Q.); 18239577551@163.com (H.W.); sp801683@aliyun.com (P.S.); yuanyuan200102@163.com (Y.X.)

**Keywords:** single-cell RNA sequencing, CDR3, autoimmune diseases, single-cell B-cell receptor sequencing

## Abstract

The combined application of single-cell RNA sequencing (scRNA-seq) and single-cell B-cell receptor sequencing (scBCR-seq) offers a multidimensional perspective for dissecting the immunopathological mechanisms of B cells in autoimmune diseases. This review systematically summarizes the principles of these techniques, the analytical framework, and their key applications in diseases such as systemic lupus erythematosus et. al. It reveals the dynamic correlations between the transcriptome of B-cell subsets and B-cell receptor (BCR) clones. Furthermore, we focus on the potential roles of dual BCR B cells and B/T biphenotypic cells in autoimmunity, emphasizing their exacerbation of disease progression through abnormal clonal expansion and autoantibody secretion. By sorting through cutting-edge advancements and bottleneck issues, this article aims to propel the innovation of multi-omics research and precision treatment paradigms for autoimmune diseases.

## 1. Introduction

Single-cell RNA sequencing, due to its high throughput, high resolution, and ability to provide comprehensive information for dissecting single-cell gene expression, can analyze the homogeneity and heterogeneity of cell development, differentiation, maturation, and effector functions at both temporal and spatial levels simultaneously [1]. It has provided unprecedented opportunities for biological and medical research and has become one of the most important tools in cellular research fields such as immunology, oncology, neuroscience, and developmental biology. It has gradually gained widespread application.

**Figure 1:**
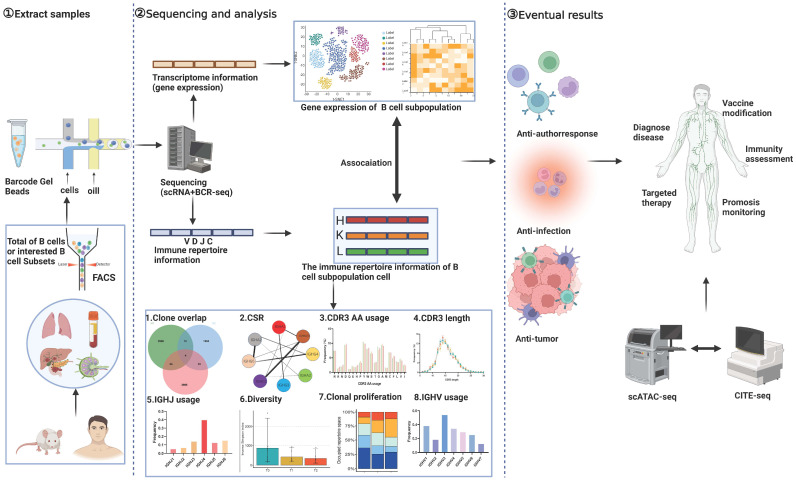
Schematic representation of scRNA + BCR-seq research workflow.

The pathogenesis of autoimmune diseases is closely related to the abnormal activation of B cells and the secretion of autoantibodies [2]. Traditional research often relies on population-level analysis, which makes it difficult to dissect the heterogeneity of B cells and their clonal dynamics. The combined application of scRNA-seq and scBCR-seq, by simultaneously capturing the transcriptome and BCR sequences of individual cells, has achieved a leap from “Separate Analysis” to “Dual-Omics Correlation Research, providing a novel tool for revealing the molecular characteristics and functional mechanisms of disease-specific B-cell subsets. The technical workflow, analysis process, and applications of studying B cells using scRNA + BCR-seq are summarized in Figure 1. In addition, this article highlights two special B-cell populations that may bring a turning point to the progression of research and treatment of autoimmune diseases. Currently, the emergence of these two B-cell subsets has not garnered sufficient attention from researchers, and single-cell sequencing technology stands as the optimal technical support for dissecting the roles of these B-cell subsets in diseases.

(1) Extract samples: The initial phase involves the meticulous preparation of research samples comprising both human and mouse B cells, and we can utilize Fluorescence-Activated Cell Sorter (FACS) to select either total B cells or B-cell subsets of interest for single-cell sequencing. This step ensures the integrity and representativeness of the cellular material for downstream analyses. (2) Sequencing and analysis: We can obtain data from scBCR-seq and scRNA-seq for each B cell. scRNA-seq: This component delves into the intricacies of B cell subsets, unraveling their unique transcriptional signatures and transcription factor profiles. scBCR-seq: Concurrently, scBCR-seq dissects the BCR repertoire, providing insights into the diversity and specificity of B cell receptors. scRNA + BCR-seq Integration: By integrating both methodologies, the correspondence between B cell subsets and their antigen-specific responses is elucidated, revealing their response characteristics to various antigens. (3) Eventual results: The synergistic power of scRNA + BCR-seq transcends traditional boundaries. It enables a comprehensive understanding of B cell function in diverse contexts, including anti-infection, antitumor, and autoimmune responses. Furthermore, when combined with cutting-edge techniques such as single-cell ATAC-seq (for chromatin accessibility) or single-cell CITE-seq (for simultaneous detection of proteins and transcripts), this approach opens up a vast array of applications in disease diagnosis, monitoring disease progression, personalized treatment strategies, immune status assessment, and even the optimization of vaccine designs.

## 2. Introduction to scRNA + BCR-Seq Technology and Its Advantages

1. scRNA-seq: scRNA-seq enables a comprehensive analysis of mRNA transcriptome characteristics at the single-cell level. Compared with traditional mRNA-Seq (Messenger RNA-sequencing) [3], which performs whole-transcriptome analysis on a population of cells, scRNA-seq offers a more refined perspective, comprehensively dissecting gene expression changes in cells across different differentiation states and microenvironments. With the capability to sequence more than 5,000 cells per sample in a single run, scRNA-seq reveals the temporal and spatial heterogeneity of multiple cell types within the study subject at a holistic level. It provides detailed information on the expression of transcription factors, cytokines, and cytokine receptors in each cell subset, allowing for comparative analysis of their functional characteristics, differentiation trajectories, and intercellular interactions [4,5]. The technical workflow of scRNA-seq includes the preparation of single-cell research samples; RNA (Ribonucleic Acid) reverse transcription; cDNA (Complementary Deoxyribonucleic acid) synthesis; construction of single-cell libraries; high-throughput sequencing; and data analysis [6]. With the rapid development and maturation of quality control techniques and analytical tools, as well as the decrease in sequencing costs, scRNA-seq has now been widely applied in biological and medical research.
2. scBCR-seq: scBCR-seq is a technology focused on analyzing the sequence characteristics of B-cell receptors from individual B cells. Compared with traditional lineage analysis techniques for the CDR3 (Complementarity-Determining Region 3) region of BCRs and next-generation sequencing technology [7], scBCR-seq provides a comprehensive analysis of the pairing of heavy and light chains in each B cell within the research samples, enabling functional analysis of the antigen epitopes corresponding to the BCR CDR3. This has brought new breakthroughs in the study of the development, differentiation, maturation, response, and tolerance of adaptive B cells. The technical workflow of scBCR-seq is consistent with the standard process of scRNA-seq, except that primers targeting the C region of B-cell heavy and light chains are used for mRNA capture and library construction. Data analysis employs specialized CellRanger software (version 7.1.0) to analyze the sequence composition and pairing of V(D)J(C) regions.
3. Prominent Advantages of scRNA + BCR-seq in Analyzing B Cells: The combination of scBCR + RNA-seq technology leverages the strengths of both sscRNA-seq and scBCR-seq, providing a novel technical approach for in-depth research on the effects and mechanisms of adaptive B-cell responses and clinical applications. It offers comprehensive information on the mRNA (Messenger Ribonucleic Acid) expression of regulatory and effector molecules during the differentiation, development, maturation, and response processes of various B-cell subsets at both temporal and spatial levels. Simultaneously, it provides characteristics of corresponding B-cell V(D)J(C) recombination and CDR3 repertoire, thereby enabling a comprehensive understanding of the BCR-antigen epitope correspondence features of various B-cell subsets at a large scale, assessing their antigen-binding capabilities, and dissecting the molecular mechanisms involved in regulation and effector functions (Figure 1).

Currently, platforms such as the 10x Genomics Chromium single-cell platform and the BD Rhapsody platform are capable of performing scRNA + BCR-seq on 1000–20,000 cells per sample, covering most single B-cell population types within a single research sample [8,9]. These technologies are gradually being adopted, and their advantages primarily include the ability to reassess and gain deeper insights into the more detailed homogeneity and heterogeneity among B-cell subsets; the discovery of new genotype-phenotype associations; the identification of novel target molecules involved in B-cell differentiation, development, response, tolerance, senescence, and other processes; the study of the entire process regulating B-cell clonal formation, evolution, and expansion; and the examination of the roles and developmental paths of rare B-cells. The scBCR + RNA-seq technology provides a multidimensional perspective for deepening the understanding of the role of B-cells in immune responses, contributing to advancements in adaptive B-cell research.

## 3. Studying B Cells in Autoimmune Diseases with scRNA + scBCR-Seq: From “Separate Analysis” to “Dual-Omics Correlation Research”

The response effects and mechanisms of B cells to self-antigens serve as the foundation for the treatment and diagnosis of autoimmune diseases. The new insights provided by scRNA-seq and scBCR-seq technologies into the characteristics of autoreactive B cells offer novel research avenues and technical means for the prevention and treatment of autoimmune diseases.

Systemic lupus erythematosus (SLE) is an autoimmune condition where autoantibodies cause damage to various tissues in the body, and the relationship between B lymphocytes and the onset and progression of SLE is extraordinary [10]. In 2021, Zheng et al. found that there is a bias in the usage of V(D)J genes for T-cell receptor (TCR) and B cell receptor (BCR) between healthy controls and SLE patients. The healthy group showed a preference for the use of *TRAV40* (T Cell Receptor Alpha Variable) and *IGHV3-21*, whereas SLE patients preferred *TRAV16* and *IGHV3-23* [11]. In 2022, Gao and others discovered the naive B cells in SLE patients exhibited upregulation of the type I interferon signaling pathway and reduction in the IL-4 signaling pathway, leading to a deviation in B-cell developmental trajectories from the normal germinal center-directed pathway towards an atypical memory B-cell pathway. Furthermore, an increase in IL-4R−IFN-β+ B cells was associated with autoantibody production and disease activity [12]. Wu et al. studied patients with SLE from two ethnic groups (Han and Tibetan) and found that Tibetan SLE patients had higher TCR abundance compared with healthy controls. Both Han and Tibetan SLE patients exhibited higher BCR abundance than the control group, indicating that the disease also exhibits differences between different ethnic groups [13]. Epstein–Barr virus (EBV) is a widely studied virus that has been linked to the pathogenesis of multiple autoimmune diseases, and it is also closely associated with SLE [14]. Hu et al. investigated the dynamic changes in immune cells and BCR characteristics of patients with SLE when co-infected with Epstein–Barr virus (SLE-EBV+). They found widespread activation in T cells, monocytes, and B cells of SLE-EBV+ patients, which is associated with enhanced autoimmune responses in SLE. The *IGHV3*(Immunoglobulin Heavy Variable) and *IGHV4* families dominate in SLE patients, with *IGHV4-34* (associated with autoreactivity) expressed at higher levels in SLE-EBV− patients but decreased in SLE-EBV+ patients. The proportion of IgM (Immunoglobulin M) was significantly reduced in SLE-EBV+ patients, while the proportions of IgG1 (Immunoglobulin G1) and IgA1 (Immunoglobulin A1) increased, suggesting that EBV infection may induce antibody class switching and enhance humoral immune responses. In addition, pseudotime trajectory analysis showed that Tregs in SLE-EBV+ patients are at an early stage of differentiation, potentially influencing disease progression by regulating other T-cell subsets [15]. Single-cell technologies have provided new insights into understanding the interaction between SLE and EBV infection. These technologies are also being utilized in other autoimmune diseases. Kawasaki disease (KD) is an autoimmune disease primarily affecting children, which can lead to multi-systemic inflammatory responses, with the cardiovascular system being the most severely affected [16]. Wang et al. discovered that in KD patients who received intravenous immunoglobulin (IVIG) treatment, despite a reduction in the total proportion of B cells, the proportion of plasma cells rises significantly. This was accompanied by oligoclonal expansion of BCR and IgG/IgA (Immunoglobulin G/Immunoglobulin A) isotype switching, suggesting enhanced B cell activation and antibody responses. Furthermore, effector memory T cells among CD8+ T cells decreased during the acute phase, and oligoclonal expansion of TCR also occurred after treatment, indicating the involvement of antigen-specific T cell responses. These findings support the notion that KD may be driven by adaptive immune responses targeting specific conventional antigens rather than superantigen mechanisms [17]. Psoriasis manifests as a chronic inflammatory dermatosis characterized by immune-mediated pathophysiology and epidermal hyperplasia [18]. In 2023, Liu et al. found that psoriasis patients exhibited lower diversity in *IGHA1* (Immunoglobulin Heavy Constant Alpha 1) and *IGHG1* (Immunoglobulin Heavy Chain Gamma 1), along with increased usage of *IGHV*. There was an increase in CDR silent mutations in *IGHA* and rearrangements in *IGHG* among psoriasis patients, accompanied by higher BCR selection pressure in the complementarity-determining regions and framework regions of both *IGHG* and *IGHA*. This selection pressure may affect the function and diversity of B-cell receptors, thereby influencing immune system responses and disease progression [19]. Membranous nephropathy (MN) represents one of the leading causes of nephrotic syndrome (NS) in the adult population [20]. The molecular features of a specific type of primary membranous nephropathy (pMN) that is negative for podocyte autoantibodies (NEG) remain unclear. Feng et al. conducted scRNA + BCR-seq analysis on renal cells and circulating CD19 cells from pediatric patients with pMN who were NEG. They found that in NEG pMN patients, substantial alterations in both gene expression patterns and clonal proliferation were observed within memory and naive B cells. Specifically, the CD38+ naive B cell population was expanded in NEG patients and demonstrated activation-related functional properties. The IgM/IgD (Immunoglobulin M/Immunoglobulin D) to IgG1 class switch was increased in NEG patients, leading to an increase in autoantibody production, which may be related to the pathological process of pMN [21]. Anti-N-methyl-D-aspartate receptor encephalitis (NMDAR-E) represents a severe autoimmune encephalitis clinically defined by the presence of prominent neuropsychiatric manifestations [22]. Li et al. found that B cells, particularly memory B cells, in the cerebrospinal fluid (CSF) of NMDAR-E patients exhibited upregulated gene expression related to immunoregulatory functions, while B cells in peripheral blood showed enhanced gene expression associated with antigen presentation. B cells in the CSF demonstrated higher clonality and mainly expressed IgG immunoglobulins, and B cells in peripheral blood primarily expressed IgM, suggesting potential antigen class switching in the CSF. In vitro experiments revealed that non-double negative (DN) cells from NMDAR-E patients could differentiate into IgD-CD27- double negative (DN) cells and antibody-secreting cells (ASC); the process may lead to the generation of NR1-IgG antibodies. To clarify the potential involvement of DN cell subsets in NR1-IgG antibody production, additional mechanistic studies are required [23]. Pemphigus represents an autoimmune disorder characterized by blistering lesions involving the skin and mucous membranes, with its pathogenesis mainly mediated by autoantibodies targeting desmoglein (Dsg) proteins [24]. Duan et al. identified and classified different cellular subsets, including B cells and T cells, in oral mucosal lesions and peripheral blood of patients with pemphigus vulgaris (PV). They observed the activation status and molecular changes in specific cellular subsets in PV patients, such as upregulated gene expression related to inflammatory responses and antigen presentation in the mucosal lesion areas. The IL-1α signaling pathway was found to contribute to the pathogenesis of PV, particularly near epithelial cells in the mucosal lesion areas. Utilizing scBCR + TCR-seq, they identified specific TCR and BCR clonotypes associated with the disease in PV patients, which contributed to understanding the immune response characteristics and potential autoreactivity in PV [25]. The aforementioned utilization of single-cell sequencing in autoimmune disease research is systematically compiled in Table 1.

While most studies have utilized scRNA-seq and scBCR-seq technologies to investigate autoimmune diseases, the research remains at the level of “Separate Analysis”, where scRNA-seq and scBCR-seq are often presented separately, lacking direct correlation at the single-cell level. scRNA-seq can identify B cell subsets (such as plasma cells, memory B cells, and regulatory B cells) and abnormally activated pathways (like IFN signaling and the NF-κB pathway). scBCR-seq allows for tracking B cell clonal expansion, somatic hypermutation (SHM), antigen selection pressure, and identifying autoreactive BCR characteristics (such as VH gene bias and CDR3 charge properties). Integrated analysis of both can correlate BCR clonotypes with cellular states (e.g., the relationship between specific clonal expansion and inflammatory phenotypes).

The correlation studies using dual-omics (scRNA-seq + scBCR-seq) have become particularly important. For instance, Nickerson et al. utilized scRNA-seq combined with scBCR-seq to uncover the high heterogeneity of age-associated B cells (ABCs) in SLE, including their dependence on Toll-like receptor (TLR) signaling and reactivity to self-antigens. ABCs not only self-renew but also differentiate into plasma cells or germinal center B cells. Furthermore, it was also found that ABCs share clonal mutations with plasma cells, indicating their significant dynamic and circular characteristics in the autoimmune responses of SLE [26]. Akama-Garren et al. found that the spleens of SLE-susceptible mice exhibited significant expansion of lymphocytes and myeloid cells, particularly an increase in the number of plasma cells, accompanied by a decrease in marginal zone B cells. BCR clonal analysis revealed that B cell clones expanded towards the plasma cell lineage, and B cells from SLE-susceptible mice demonstrated a higher frequency of somatic hypermutation [27]. Focusing on the transcriptional state of B cells or certain B cell subsets while also integrating their immune repertoire characteristics, it provides deeper insights into the pathogenesis and potential therapeutic strategies of autoimmune diseases. This “Dual-Omics Correlation Research” has also been investigated in other autoimmune diseases. Inflammatory Bowel Disease (IBD) is a chronic inflammatory autoimmune disorder of the digestive tract, primarily encompassing two types: Crohn’s Disease (CD) and Ulcerative Colitis (UC) [28]. Boland et al. revealed the clonal relationships and heterogeneity of immune cells in UC patients, particularly noting a significant increase in IgG1+ plasma cells in the colonic tissue of UC patients. These cells exhibited unique transcriptional features and BCR clonotypes. In healthy states, BCR clonotypes are shared among multiple plasma cell clusters, but in UC patients, specific plasma cell clusters (such as B1 plasma cell clusters) showed increased clonal expansion, providing new insights for future therapeutic interventions [29]. Rheumatoid arthritis (RA) is an autoimmune disease with synovitis as its pathological basis, which ultimately may lead to joint deformity [30]. Dunlap et al. analyzed B cells in synovial tissue and peripheral blood of RA patients. They revealed that non-naive B cells enriched in the synovium, including NR4A1+ activated B cells, plasma cells, and age-associated B cells, exhibited higher somatic mutation rates compared with B cells in peripheral blood. Notably, NR4A1+ activated B cells shared clones with age-associated B cells, and naive MT+ B cells shared clones with both age-associated B cells (ABCs) and activated B cells, suggesting that naive MT+ B cells may represent the precursors of ABCs and activated B cells in the synovium. This provides a new perspective for understanding the immunopathological mechanisms of RA [31]. Xu et al. utilized dual-omics (scRNA-seq + scBCR-seq) to discover that pemphigus lesion-associated antibody-secreting cells (ASCs) undergo clonal expansion and accumulate IgG4 class switching within pemphigus lesions. Furthermore, all ASC immunoglobulin genes exhibited highly variable somatic hypermutation (SHM). ASCs acquired pathogenicity and Dsg autoreactivity through aberrant VDJ recombination in the bone marrow or selection mediated by SHM in germinal centers. Significant expansion and heterogeneity were observed within T cell populations, with CXCL13+IL-21+Th17 cells appearing to promote B cell antibody responses and recruitment [32]. Neuromyelitis Optica Spectrum Disorder (NMOSD) is an uncommon autoimmune disease [33]. Jiang et al. found that patients with NMOSD exhibited an increased proportion of *IGHG* and *IGHA* compared with healthy controls. BCR clonality assessment revealed a slight increase in the percentage of clonal BCRs in NMOSD patients after steroid treatment, primarily consisting of IgA and IgG subtypes. The usage of *IGHV* genes showed differences in NMOSD patients but was not significantly different from healthy controls. The number of clonal BCRs in naive B cells, plasma cells, and memory B cells increased significantly. Overall, NMOSD patients displayed transcriptional homogeneity and extensive BCR expansion during the acute phase, suggesting that the B cell response is mainly focused on specific antibodies rather than a response to superantigens [34]. Primary Sjögren’s syndrome (pSS) is a systemic, chronic autoimmune disease characterized by progressive exocrine gland damage and lymphocyte proliferation [35]. Arvidsson et al. analyzed the B cell composition and BCR gene usage in pSS patients, stratified according to their autoantibody status against Sjögren’s syndrome antigen A/B (SSA/SSB). They found that compared with healthy controls, there were significant changes in the B cell subset composition in pSS patients, particularly in SSA/SSB double-positive patients, where a lower proportion of memory B cells were observed and a higher proportion of naive B cells. All pSS patient groups showed upregulation of interferon (IFN) response genes in their B cell subsets compared with controls, with the most significant upregulation in the SSA/SSB double-positive patient group. Additionally, a higher proportion of unmutated VDJ transcripts were present in memory B cells of pSS patients, especially in SSA/SSB double-positive patients, suggesting that the somatic hypermutation process may be disturbed [36]. Thyroid-associated ophthalmopathy (TAO) is an organ-specific autoimmune disease characterized by infiltrative lesions in the retrobulbar and periorbital tissues [37]. Li et al. conducted an in-depth analysis of the peripheral immune characteristics of patients with TAO. During the active phase of TAO, regulatory B cells (Bregs) were significantly decreased. Further cell differentiation trajectory analysis revealed that the transition of B cells towards the Breg phenotype might be impaired during the active phase of TAO. Compared with the inactive phase and normal controls, B cell clonotypes in patients with active TAO were likely more diverse. Additionally, the diversity of CDR3 increased significantly, potentially reflecting an adaptive immune response of B cells to multiple antigens. The decreased proportion of Breg cells coupled with increased BCR diversity may indicate an impaired role of Breg cells in regulating B cell responses and maintaining immune balance [38]. Myasthenia gravis (MG) is an autoimmune disorder resulting from disrupted communication at the neuromuscular junction [39]. Tian et al. observed the B-cell lineage reconstitution and dynamic changes in chimeric antigen receptor (CAR-T) cells targeting B-cell maturation antigen (BCMA) in patients with Myasthenia gravis (MG). The study revealed significant clonal expansion of B cells, particularly plasma cells and plasmablasts (PC/PBs), seen prior to treatment. indicating abnormal activation and expansion of B cells in MG patients. After treatment, there were notable changes in the B-cell population, particularly the observation that most of the newly formed B cells were unactivated naive B cells. Furthermore, a continuous reduction in autoantibodies was observed in patients 18 months after treatment. These discoveries shed new light on the use of CAR-T cell therapy for autoimmune diseases and also give valuable direction for enhancing CAR-T cell therapy in the future [40]. Ye et al. analyzed peripheral blood B cells from patients with anti-melanoma differentiation-associated gene 5-positive dermatomyositis (MDA5+ DM), a disease control group with idiopathic inflammatory myopathies (IIM), and healthy donors. The study found that during the active phase of MDA5+DM, patients had a higher proportion of memory B cells, ASCs, and interferon-stimulated gene-positive B cells (ISG+ B cells), which decreased during the remission phase. Flow cytometry validation showed an increased frequency of ASCs in patients with active MDA5+ DM. Single-cell BCR sequencing analysis revealed clonal expansion and autoimmune-prone characteristics of these ASCs, particularly antigen-driven selection of *IGHG* and *IGHA*. The results indicate that B-cell responses are significantly activated in patients with active MDA5+ DM, with enhanced terminal differentiation of ASCs [41]. “Dual-Omics Correlation Research” provides a valuable research approach for studying autoimmune diseases by simultaneously investigating the transcriptome and immune repertoire of specific cell subpopulations involved in the disease. This approach reveals the diversity of cellular states, clonal expansion trajectories, and the dynamic associations between receptors and the transcriptome. The aforementioned utilization of single-cell sequencing in autoimmune disease research is systematically compiled in Table 2.

## 4. ScRNA + scBCR-Seq and New Directions in B Cells in Autoimmune Diseases: Dual BCR B Cells and B/T Biphenotypic Cells

Despite significant advancements in single-cell technologies, they have not yet been fully leveraged in current research. However, studies on dual BCR B cells in autoimmune diseases can now be conducted. The discovery of dual receptor B cells dates back to 1961 [42]. However, for many years, the VDJ gene rearrangement in B cells has remained unclear [43,44,45,46]. There has been little attention paid to dual BCR B cells, but the emergence of this new B-cell subset cannot but draw our attention [47,48]. In 2014, it was reported that receptor editing is one of the main mechanisms of B-cell tolerance; however, it may also result in the inclusion of both alleles at the immunoglobulin light chain locus, leading to B cells that produce two distinct immunoglobulin light chains at the same time. These cells often co-express autoreactive and non-autoreactive antibodies, akin to a “Trojan horse,” since the presence and activity of non-autoreactive antigen receptors might facilitate their development, stimulation, and specialization into effector cells that also produce and release autoantibodies. Studies have shown that in certain autoimmune mouse models, dual κ-chain B cells significantly expand into effector B-cell subsets, suggesting that they might be significantly involved in disease pathogenesis [49]. In 2019, Shi et al. employed scBCR-seq sequencing to examine V(D)J recombination patterns of two or more IgH or IgL chains in hundreds to thousands of individual B cells. Moreover, each Ig class displayed a distinct V_H_DJ_H_ recombination pattern within single B cells that expressed multiple Ig classes [50]. In 2023, Zhu et al. also utilized scBCR-seq to discover that approximately 10% of B cells in human peripheral blood and bone marrow express dual (or multiple) BCRs, while the proportion reaches about 20% in mouse bone marrow and peripheral blood memory B cells. Additionally, in each sample, there were individual B cells containing mRNA for three or more functional Ig heavy or light chains. Furthermore, the study emphasized the necessity of classifying and analyzing B cell repertoires based on single, dual (or multiple) BCR B cells (i.e., H + L/H + K/H + K + L/H + L1 + L2/H + K1 + K2/H1 + H2 + K + L, etc.) [51]. To date, research on dual TCR T cells in various diseases has been progressively conducted. Xu Y et al. also discovered the existence of dual TCR T cells in ankylosing spondylitis through scRNA + TCR-seq and stratified the presence of dual TCR T cells into T cell subsets, revealing the participation of dual TCR Treg and pTh17 T cells in the autoimmune response in ankylosing spondylitis [52]. Peng et al. also provided a more in-depth explanation of the underlying mechanisms of the response against tumors by tumor-infiltrating T lymphocytes within the tumor microenvironment (TME). By analyzing a large amount of scTCR + RNA-seq data, they discovered a significant presence of dual TCR T cells within the TME. These cells are characterized by their clonal expansion capabilities and striking migratory abilities across distinct tissues, including normal tissues, blood, and tissues surrounding tumors in patients with non-small cell lung cancer. Significantly, dual TCR CD8+ T cells are mainly part of the CXCL13+ subset, demonstrating strong anti-cancer activity and a high likelihood of being found in tumor tissues. This suggests that dual TCR T cells constitute a “unique subset of TILs” in the antitumor response [53]. Xu and others revealed the significant function of dual TCR T cells in Kawasaki disease, discovering that the proportion of these cells rose markedly after IVIG treatment and exhibited clonal expansion and preferential V gene usage, particularly within CD8 T and Treg cells. The findings indicate that dual TCR T cells may contribute to the pathogenesis of Kawasaki disease and the immunomodulation following IVIG treatment, providing new insights into understanding the disease process and improving treatment [54]. Research on dual BCR B cells also has a basis. Yuanning Yao et al., in a further analysis of the scRNA + BCR-seq data from Gong et al., found that dual BCR B cells exist both in the TME and inflammatory environments, primarily originating from memory B cells. The main paired dual BCR type was H + K + λ. They also discovered that *IGHV1-46* was relatively highly utilized in dual receptor B cells, and there was a certain proportion of overlapping CDR3 sequences among dual receptor B cells across different nasopharyngeal carcinoma (NPC) patients. These discoveries indicate that dual receptor B cells might contribute to the anti-cancer response in nasopharyngeal carcinoma [55]. However, research on dual receptor B cells in autoimmune diseases has also been conducted previously, although single-cell sequencing technology was not utilized at that time. For instance, in SLE, dual receptor B cells exhibit autoreactivity more frequently than single receptor B cells. Dual-reactive B cells are predominantly found in plasmablast and memory B cell subsets, suggesting they may play a significant role in autoimmune diseases [56]. Fraser et al. found that the proportion of B cells expressing both κ and λ light chains simultaneously was significantly increased in SLE patients, whereas it was less common in healthy controls and patients with other systemic autoimmune diseases (such as granulomatosis with polyangiitis) [57]. Furthermore, in SLE, dual receptor B cells are more sensitive to Toll-like receptor 7/9 stimulation and type I/II interferons and rely on IL-21 to maintain their homeostasis. Dual receptor B cells exhibit higher levels of MHC II and co-receptor expression, have stronger proliferative capacity, and generate more robust memory responses upon T cell-dependent antigen stimulation [58]. In 2022, Peterson et al. found that approximately 25% of SLE patients had abnormally elevated frequencies of dual receptor B cells in their blood circulation. However, this phenomenon was not due to an actual increase in dual receptor B cells but rather because the B cells of these patients were modified by *VH4-34* autoantibodies, which bind to B cell-specific surface antigens [59]. Dual receptor B cells exhibit autoreactivity in SLE and may participate in the production of autoantibodies, exacerbating the disease pathology. The relationship between dual receptor B cells and autoimmune diseases cannot be overlooked. Currently, we can study dual BCR B cells in autoimmune diseases through the combination of scRNA-seq and scBCR-seq, accurately identifying the transcriptional status and BCR characteristics of dual BCR B cells and revealing their clonal expansion trajectories and autoreactive potential. The study of dual BCR B cells in autoimmune diseases may be a research trend in the future, and this special cell subset warrants our focused attention.

In addition to the dual BCR B cell subset, we can also study B/T biphenotypic cells using single-cell technology. The development of T and B cells is usually strictly distinguished, and they can be differentiated by markers such as CD19, CD20, CD3, TCR, and BCR. However, with ongoing exploration in immunology, some cells have been found to co-express TCR and BCR, and the existence of these cells has been verified at the protein level. This group of cells also plays a crucial role in disease. B/T biphenotypic cells were initially discovered in HIV (Human Immunodeficiency Virus)-infected individuals. In 1990, Landay A. et al. used flow cytometry to detect CD3+ T cells expressing CD20 in the peripheral blood of HIV-infected individuals. Due to the technological limitations at the time, this discovery could not be further pursued [60]. Subsequent extensive research has shown the expression of B-cell markers in peripheral T-cell lymphomas [61,62,63,64]. This indicates the initial existence of B/T biphenotypic cells. Research on B/T biphenotypic cells in autoimmune diseases has also been conducted to some extent. In 2011, Eggleton P et al. found a high proportion of CD20-expressing Th17 cells in RA patients and suggested that this population of cells could be another target for anti-CD20 therapy [65]. In 2014, Palanichamy et al. found some T cells expressing CD20 in MS (Multiple Sclerosis) patients and clearly validated the existence of CD20+ CD3+ cells through flow cytometry. In MS patients, CD20+ CD3+ biphenotypic cells were effectively depleted by rituximab (RTX) treatment, but the specific role of these cells in MS remains to be studied [66]. However, the depletion effect of RTX on this group of cells was also demonstrated in another study. In 2016, Alunno et al. showed in patients with primary Sjögren’s syndrome that some IL-17-producing T cells, including CD4+ Th17 cells and double-negative (DN) T cells, also express the CD20 molecule. These CD20+ Th17 cells were depleted under RTX treatment, revealing that RTX may exert its effect in pSS treatment by directly targeting these T cells [67]. In 2018, Niu et al. found that CD3⁺CD20⁺ T cells in the peripheral blood of psoriasis patients exhibited an activated effector phenotype during the disease and were capable of producing more pro-inflammatory cytokines (such as IL-17A, TNF-α, and IL-21), which positively correlated with disease severity. The results suggest that CD20⁺CD3⁺ T cells may contribute to the pathogenesis of psoriasis [68]. A recent study on type 1 diabetes (T1D) and BCR + TCR+ biphenotypic cells has attracted widespread attention. Ahmed et al. found that this population of cells is significantly expanded in T1D patients and simultaneously expresses TCR and BCR. A public clonotype (x-clonotype) exists in the BCR of B/T cells; the peptide encoded by it (x-Id peptide) can form stable complexes with DQ8 molecules and strongly activate autoreactive CD4 T cells in T1D patients but has no such effect on healthy controls. Additionally, antibodies secreted by B/T cells (x-monoclonal antibody) can also activate insulin-specific CD4 T cells, suggesting that B/T cells may participate in the pathogenesis of T1D through dual mechanisms involving both BCR and TCR. These findings reveal the potential role of BCR+TCR+ cells in the pathogenesis of T1D and provide new directions for the development of new diagnostic biomarkers and therapeutic strategies [69]. The advancement of single-cell sequencing technology has facilitated research into this group of cells. In 2025, Zhang et al., through single-cell sequencing and flow cytometry analysis, found that biphenotypic cells expressing lineage markers of both T and B cells are widely distributed in the bone marrow, lymph nodes, spleen, and peripheral blood of mice. These biphenotypic cells may originate from pre-B cells and can be activated through TCR and BCR signaling pathways, exhibiting dual functional characteristics of both T and B cells. For instance, under antigen-specific stimulation, they can secrete higher levels of IL-2 but lower levels of TNF-α and participate in humoral and cellular immune responses after vaccination. Additionally, similar B/T biphenotypic cells were detected in human peripheral blood, albeit at a lower frequency compared with mice, but they similarly demonstrated responsiveness to TCR and BCR stimulation [70].These studies have set a paradigm for our research on B/T biphenotypic cells in autoimmune diseases. By leveraging single-cell transcriptome and immune repertoire sequencing data, we can identify the subpopulation characteristics, functional properties, BCR and TCR diversity of these cells, as well as their dynamic changes in immune responses. The discovery of B/T biphenotypic cells highlights the unique advantages of single-cell multi-omics in dissecting the “cross-boundary” behavior of immune cells in diseases. Future research needs to combine functional experiments to verify whether their dual signal activation directly participates in autoimmune target organ damage.

## 5. Concluding Remarks

The scRNA + BCR-seq technology, by analyzing the transcriptome and BCR clonal characteristics of individual B cells, has redefined the heterogeneity of B cells in autoimmune diseases and provided potential molecular markers for targeted therapy (such as the preferential usage of IGHV3-23 in SLE). However, current research still faces challenges such as insufficient experimental validation and high technical costs. Future studies need to integrate multi-omics technologies such as scATAC-seq (Single-Cell Assay for Transposase-Accessible Chromatin using sequencing) and spatial transcriptome sequencing to reveal the synergistic mechanisms of epigenetic regulation and microenvironment interaction. In addition, functional validation of new subsets such as dual BCR B cells is urgently needed, and whether their dual receptor signaling directly drives autoimmune damage requires further exploration. Through interdisciplinary collaboration and technological innovation, scRNA + BCR-seq has the potential to accelerate the process of precision diagnosis and treatment of autoimmune diseases.

## Figures and Tables

**Table 1:** Application of single-cell sequencing technology in autoimmune diseases: separate analysis.

Disease	Reference	Sequencing Technology	Significance
Systemic lupus erythematosus (SLE)	Zheng et al. [11]	ScBCR-seq; scTCR-seq	Provide a new approach for diagnosis and treatment of SLE
Systemic lupus erythematosus	Gao et al. [12]	ScRNA-seq; scBCR-seq	Reveal the changes in B-cell developmental characteristics in diseases.
Systemic lupus erythematosus	Wu et al. [13]	ScRNA-seq; ScBCR-seq; scTCR-seq	Reveal the immune cell heterogeneity in Han and Tibetan SLE patients.
Systemic lupus erythematosus	Hu et al. [15]	ScRNA-seq; scBCR-seq	Understand the immune response mechanisms to EBV infection in patients with SLE.
Kawasaki disease	Wang et al. [17]	ScRNA-seq; scBCR-seq ScTCR-seq	To elucidate the immune response mechanisms of diseases.
Psoriasis	Liu et al. [19]	ScRNA-seq; scBCR-seq	Reveal the immune response mechanisms of B cells in psoriasis patients.
Primary membranous nephropathy	Feng et al. [21]	ScRNA-seq; scBCR-seq	Reveal the mechanism of immune response
Anti-N-methyl-D-aspartate receptor encephalitis	Li et al. [23]	ScRNA-seq; scBCR-seq	Reveal the mechanism of immune response
Pemphigus vulgaris	Duan et al. [25]	ScRNA-seq; scBCR-seq; ScTCR-seq	Reveal the immune response characteristics and potential autoreactivity of PV.

**Table 2:** Application of single-cell sequencing technology in autoimmune diseases: dual-omics correlation research.

Disease	Reference	Sequencing Technology	Significance
Systemic lupus erythematosus	Nickerson et al. [26]	ScRNA-seq; scBCR-seq	Reveal the direct role of age-associated B cells in the pathogenesis of SLE.
Systemic lupus erythematosus	Akama-Garren et al. [27]	ScRNA-seq; scBCR-seq	Provide new insights into the pathogenesis of diseases.
Ulcerative colitis	Boland BS et al. [29]	ScRNA-seq; scBCR-seq	Reveal the mechanism of immune response
Rheumatoid arthritis	Dunlap et al. [31]	ScRNA-seq; scBCR-seq	Provide new insights into understanding the immunopathological mechanisms of RA.
Pemphigus	Xu et al. [32]	ScRNA-seq; scBCR-seq ScTCR-seq	Elucidate the pathogenesis of pemphigus skin lesions.
Neuromyelitis optica spectrum disorder	Jiang et al. [34]	ScRNA-seq; scBCR-seq	Reveal the mechanism of immune response
Primary sjögren’s syndrome	Arvidsson et al. [36]	ScRNA-seq; scBCR-seq	Reveal the mechanism of immune response
Thyroid-associated ophthalmopathy	Li et al. [38]	ScRNA-seq; scBCR-seq	Explore the pathogenesis and treatment of TAO.
Myasthenia gravis	Tian et al. [40]	ScRNA-seq; scBCR-seq	Improve CAR T-cell immunotherapy in autoimmune diseases.
Anti-melanoma differentiation-associated gene 5-positive dermatomyositis	Ye et al. [41]	ScRNA-seq; scBCR-seq	Reveal the key immunopathogenic characteristics of MDA5+ DM.

## Data Availability

Not applicable.
